# Prevalence and impact of visual aura in migraine and probable migraine: a population study

**DOI:** 10.1038/s41598-021-04250-3

**Published:** 2022-01-10

**Authors:** Kyung Min Kim, Byung-Kun Kim, Wonwoo Lee, Heewon Hwang, Kyoung Heo, Min Kyung Chu

**Affiliations:** 1 Department of Neurology, Severance Hospital, Yonsei University College of Medicine, 50-1 Yonsei-ro, Seodaemun-gu, Seoul, 03722 Republic of Korea; 2 Department of Neurology, Nowon Eulji Medical Center, Eulji University School of Medicine, Seoul, Republic of Korea

**Keywords:** Diseases, Neurology, Signs and symptoms

## Abstract

Visual aura (VA) presents in 98% of cases of migraine with aura. However, data on its prevalence and impact in individuals with migraine and probable migraine (PM) are limited. Data from the nation-wide, population-based Circannual Change in Headache and Sleep Study were collected. Participants with VA rating scale scores ≥ 3 were classified as having VA. Of 3,030 participants, 170 (5.6%) and 337 (11.1%) had migraine and PM, respectively; VA prevalence did not differ between these cohorts (29.4% [50/170] vs. 24.3% [82/337], *p* = 0.219). Participants with migraine with VA had a higher headache frequency per month (4.0 [2.0–10.0] vs. 2.0 [1.0–4.8], *p* = 0.014) and more severe cutaneous allodynia (12-item Allodynia Symptom Checklist score; 3.0 [1.0–8.0] vs. 2.0 [0.0–4.8], *p* = 0.046) than those without VA. Participants with PM with VA had a higher headache frequency per month (2.0 [2.0–8.0] vs. 2.0 [0.6–4.0], *p* = 0.001), greater disability (Migraine Disability Assessment score; 10.0 [5.0–26.3] vs. 5.0 [2.0–12.0], *p* < 0.001), and more severe cutaneous allodynia (12-item Allodynia Symptom Checklist score, 2.5 [0.0–6.0] vs. 0.0 [0.0–3.0], *p* < 0.001) than those without VA. VA prevalence was similar between migraine and PM. Some symptoms were more severe in the presence of VA.

## Introduction

Aura is a complex, reversible neurological manifestation that occurs during or before the onset of the headache phase in migraine^1^. The third edition of the International Classification of Headache Disorders (ICHD-3) has defined migraine with aura (MA) as a condition with fully reversible visual, sensory, motor, speech and/or language, brainstem, and retinal symptoms. Visual aura (VA) is the most common form of aura in MA, occurring either alone or in combination with other auras in 98% of cases^2–4^. MA differs from migraine without aura (MO) in terms of the prevalence, comorbidities, triggering factors, and clinical characteristics^5–8^.

Epidemiological studies have reported the prevalence and impact of aura in general populations^9–11^. These studies used questionnaires to identify the aura symptoms; however, most questionnaires were not validated through a comparison between their results and the doctor’s diagnosis of aura. Recently, a self-administered VA rating scale (VARS) was developed based on the typical symptoms of VA^12,13^. It showed a high sensitivity and specificity for VA, and may allow a validated investigation of VA.

Probable migraine (PM) is a subtype of migraine that fulfills all but one criterion of migraine^1^. Its symptoms and associated disability differ from that of migraine, with a typically shorter attack duration, milder headache intensity, and lesser anxiety and depression^14,15^. PM affects 5%–15% of the general population annually^16^, and aura has been reported as a manifestation^17^. Although VA is a common symptom of migraine, reports of its impact on the clinical presentations of migraine and PM are currently scarce. If the clinical presentations of migraine and PM significantly differ according to the presence of VA, both conditions can be managed with more ease. Furthermore, no study has reported the prevalence of VA in individuals with PM in a general population-based setting. We hypothesized that the prevalence of VA in PM is similar to that in migraine, and the accompaniment of VA affects the clinical features of PM as it does of migraine. The present study aimed to compare the prevalence and impact of VA between PM and migraine using a nation-wide population-based sample in Korea.

## Methods

### Survey

The Circannual Change in Headache and Sleep (CHASE) study was a nation-wide, population-based survey that was conducted in October 2020 to evaluate the circannual changes in headache and sleep. The study had a baseline assessment phase, followed by longitudinal assessments performed every 3 months for over a year. At the baseline assessment, the study included modules for the diagnosis of headache, depression, anxiety, insomnia, widespread pain, and obstructive sleep apnea. It also comprised modules for disability and impact of headaches, effects of acute headache treatment, quality of sleep, stress, and physical activity.

We used a two-stage, clustered random sampling method proportional to the population distribution of all Korean territories, except Jeju-do, based on the national population survey data of 2015. The target sample size was 3,000 and the estimated sampling error was ± 1.8%. We conducted a web-based survey among adults aged 20–59 years with technical support from Hankook Research. This study used data collected during the baseline assessment phase of the CHASE study.

### Migraine and PM diagnosis

Migraine was diagnosed based on the following diagnostic criteria for MO in the ICHD-3 (code 1.1): A. At least five attacks fulfilling criteria B–D; B. Headache attacks lasting for 4–72 h (when untreated or treated unsuccessfully); C. Headaches having at least two of the following four characteristics: (1) unilateral location, (2) pulsating quality, (3) moderate or severe pain intensity, and (4) aggravation by or causing avoidance of routine physical activity (e.g., walking or climbing stairs); D. Occurrence of at least one of the following during the headache: (1) nausea and/or vomiting or (2) photophobia and phonophobia; and E. Attacks not better accounted for by another ICHD-3 diagnosis^1^. The diagnostic validity was evaluated by comparing the migraine diagnoses in the survey with the doctors’ diagnoses through additional telephone interviews. The sensitivity and specificity for the diagnosis of migraine were 75.0% and 88.2%, respectively.

PM was also diagnosed based on the ICHD-3; if a participant did not fulfill only one of the five criteria for migraine, they were classified as having PM (code 1.5). If a participant met the criteria for both tension-type headache (TTH) and PM, they were classified as having TTH according to the ICHD-3.

Because the diagnosis of MA (code 1.2) was made when a participant’s headache attack fulfilled the diagnostic criteria of both MA and MO, migraine in the present study refers to both MA and MO^1^. Similarly, PM in the present study refers to both PM with aura (code 1.5.2) and PM without aura (code 1.5.1).

### VA assessment

The self-reporting VARS questionnaire was used for the assessment of VA^12,13^. This questionnaire comprises the following five visual-symptom items (each scored 1–3): (1) duration of 5–60 min (3 points), (2) develops gradually in ≥ 5 min (2 points), (3) scotoma (2 points), (4) zig-zag lines (fortification; 2 points), and (5) unilateral (homonymous; 1 point)^18^. If the sum of the scores of these five items was ≥ 3, the participant was classified as having VA. If participants were noted to have migraine as well as VA, we classified them as having “migraine with VA.” If participants were noted to have migraine, but not VA, we classified them as having “migraine without VA.” We similarly classified participants as having PM with aura and having PM without aura. The self-reporting VARS questionnaire showed a sensitivity of 96.4% and a specificity of 79.5%, when its results were compared to the doctor’s diagnosis of VA^12^.

### Impact and disability of migraine and PM

The impact of migraine and PM was assessed by the Headache Impact Test-6 (HIT-6)^19^. Disability in migraine and PM was evaluated by the Migraine Disability Assessment (MIDAS)^20^. We used the Korean versions of the HIT-6 and MIDAS, which were previously validated for the Korean language^21^.

### Assessment of cutaneous allodynia, widespread pain, and acute treatment optimization

Cutaneous allodynia (CA) was assessed by the 12-item Allodynia Symptom Checklist (ASC-12), which measured interictal CA during the month prior to migraine occurrence via various symptoms of CA^22^. Widespread pain was evaluated based on the Widespread Pain Index (WPI) in the 2016 American College of Rheumatology criteria for fibromyalgia^23^. Acute treatment optimization was assessed using the 6-item version of the Migraine-Treatment Optimization Questionnaire (M-TOQ-6)^24^. A higher score on the M-TOQ-6 indicated a better response to acute treatment^24^.

### Assessment of anxiety, depression, and stress

Anxiety and depression were evaluated by the Generalized Anxiety Disorder-7 (GAD-7) questionnaire and the Patient Health Questionnaire-9 (PHQ-9), respectively^25–27^. Stress was assessed by the Korean version of the Brief Encounter Psychosocial Instrument (BEPSI-K)^28,29^. The GAD-7 questionnaire, PHQ-9, and BEPSI-K were previously validated for the Korean language^28,30,31^.

### Ethical approval

The CHASE study was approved by the Institutional Review Board of the Severance Hospital, Yonsei University (approval no. 2020-0034-001). The participants provided written informed consent to participate in this study. All methods were performed in accordance with the Declaration of Helsinki and its subsequent amendments^32^.

### Statistical analyses

The 1-year prevalence of migraine was calculated as the number of cases per 100 persons. For continuous variables, normality was tested with the Kolmogorov–Smirnov test. After normality was confirmed, the Student’s *t* test or an analysis of variance was used when appropriate. If the normality was not confirmed, the Mann–Whitney *U* test or the Kruskal–Wallis test was used. We used the chi-square test to compare the overall and sex-specific prevalence of VA between participants with migraine and those with PM. Clinical characteristics that were expressed as continuous variables were compared using multivariable linear regression analyses after adjustments for age and sex. Conversely, clinical characteristics that were expressed as categorical variables were compared using logistic regression analyses after adjustments for age and sex. The Statistical Package for Social Sciences (version 24.0; IBM, Armonk, NY, USA) was used for all statistical analyses. The statistical significance was set to *p* < 0.05. Because our web-based survey does not register as being complete without responses to all the questions, there were no missing data in our study.

## Results

### Sample and survey

**Figure 1:**
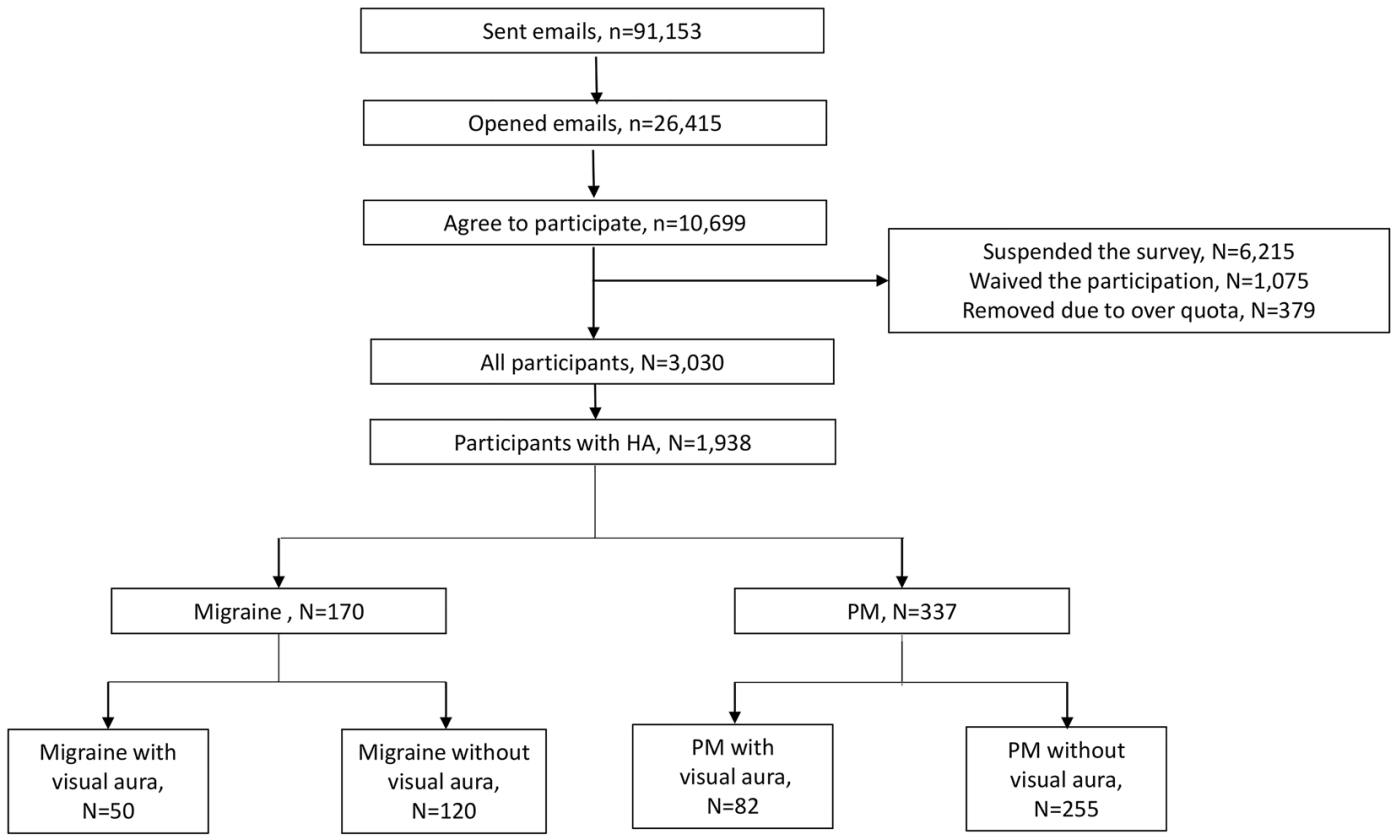
Flowchart of the participants in the study. HA: headache, PM: probable migraine.

The flow of participants throughout the study is summarized in Fig. 1. The cooperation rate in our study was 28.3% (3030/10,699). The sex, age, size of residential area, and educational level of our participants did not differ statistically significantly from that of the total population of Korea (Table 1). The survey was conducted in October 2020.

**Table 1:** Sociodemographic distributions of the survey participants, total Korean population, and cases identified as those of migraine and probable migraine.

	Survey participants N (%)	Total Korean population N (%)	*P* value	Migraine, N, % (95% CI)	Probable migraine, N, % (95% CI)
Men	1551 (51.2)	15,529,105 (51.2)	> 0.999	41, 2.6 (1.8–3.4)	130, 8.4 (7.0–9.7)
Women	1479 (48.8)	14,778,651 (48.8)		129, 8.7 (7.2–10.2)	207, 14.0 (12.5–16.3)
**Age (years)**					
20–29	673 (22.2)	6,719,119 (22.1)	> 0.999	34, 5.1 (3.4–6.7)	69, 10.3 (8.0–12.6)
30–39	685 (22.6)	6,839,377 (22.6)		44, 6.4 (4.6–8.3)	86, 12.6 (10.1–15.0)
40–49	819 (27.0)	8,208,901 (27.1)		60, 7.3 (5.5–9.1)	99, 12.1 (9.9–14.3)
50–59	853 (28.2)	8,540,359 (28.2)		32, 3.8 (2.5–5.0)	83, 10.4 (8.2–12.6)
**Size of residential area**					
Large city	1364 (45.0)	13,667,248 (45.1)	0.488	65, 4.8 (3.6–5.9)	161, 12.0 (10.3–13.8)
Medium-to-small city	1376 (45.4)	12,143,800 (40.1)		86, 6.3 (5.0–7.5)	146, 10.8 (9.1–12.5)
Rural area	290 (9.6)	4,496,708 (14.8)		19, 6.6 (3.7–9.4)	30, 10.3 (6.8–13.9)
**Education level**					
High school or less	1212 (40.0)	12,395,872 (40.9)	0.897	66, 5.4 (4.1–6.7)	131, 11.3 (9.4–13.2)
College or more	1818 (60.0)	17,911,884 (59.1)		104, 5.7 (4.7–6.8)	206, 11.3 (9.8–12.8)
Total	3030 (100.0)	30,307,756 (100.0)		170, 5.6 (4.8–6.4)	337, 11.1 (10.2–12.5)

CI: confidence interval.

### Prevalence of migraine and PM

Among the 3030 participants, 1,938 (63.7%) reported that they had experienced headaches during the previous year. Furthermore, 170 (5.6%) and 337 (11.1%) participants were classified as having migraine and PM, respectively (Fig. 1).

### Prevalence of VA among participants with migraine and PM

Among the 1,938 participants with headache, 406 (20.9%) had VARS scores ≥ 3 and were classified as having VA. Among the participants with migraine and PM, 50 (29.4%) and 82 (24.3%) were classified as having VA, respectively (Fig. 1). The prevalence of VA was similar between participants with migraine and participants with PM (*p* = 0.219).

The frequencies of VA among participants with migraine and those with PM are summarized in Table 2. The most common frequency of VA in headache attacks was 10%–24% in participants with migraine and in participants with PM. Occurrence of VA in 100% of all headache attacks was only reported in 4.0% and 1.2% of the participants with migraine and PM, respectively.

**Table 2:** Frequency of visual aura among all headache attacks in participants with migraine and probable migraine.

Frequency of visual aura in all headache attacks	Migraine, N (%)	Probable migraine, N (%)
< 10%	7 (14.0)	19 (23.2)
10–24%	18 (36.0)	29 (35.4)
25–50%	12 (24.0)	19 (23.2)
51–75%	8 (16.0)	11 (13.4)
76–99%	3 (6.0)	3 (3.7)
100%	2 (4.0)	1 (1.2)
Total	50 (100.0)	82 (100.0)

### Sex-specific prevalence of VA in migraine and PM

**Figure 2:**
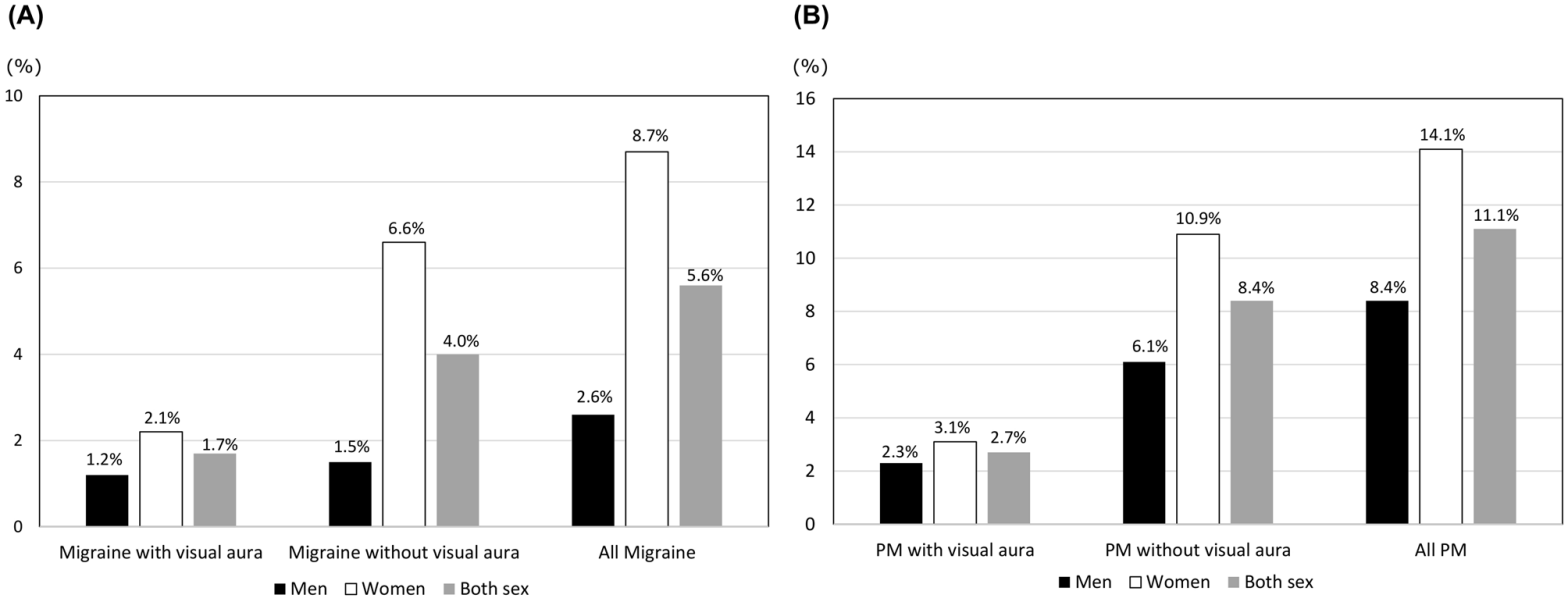
Sex-specific prevalence of visual aura in migraine (**A**) and probable migraine (**B**). PM: probable migraine.

The sex-specific prevalence of VA in participants with migraine and in those with PM is summarized in Fig. 2A and B, respectively. Among the 1,479 women analyzed, 32 (2.2%) and 46 (3.1%) participants were classified as having migraine with VA and PM with VA, respectively. Among the 1,551 men analyzed, 18 (1.2%) and 36 (2.3%) participants were classified as having migraine with VA and PM with VA, respectively. The prevalence of migraine with VA and migraine without VA showed a statistically significant difference among women (2.1% [32/1,479] vs. 6.6% [97/1,479], *p* < 0.001), but not among men (1.2% [18/1,551] vs. 1.5% [23/1,551], *p* = 0.886). However, the prevalence of PM with VA and PM without VA showed a statistically significant difference in both men (2.3% [36/1,551] vs. 6.1% [94/1,551], *p* < 0.001) and women (3.1% [46/1,479] vs. 10.9% [161/1,479], *p* < 0.001).

The female-to-male ratio for the prevalence of migraine with VA (1.86) was statistically significantly lower than that for the prevalence of migraine without VA (4.42) (*p* = 0.019). However, the female-to-male ratio for the prevalence of PM did not differ statistically significantly between participants with PM with VA (2.61) and those with PM without VA (3.50, *p* = 0.256).

### Age-specific prevalence of VA in migraine and PM

**Figure 3:**
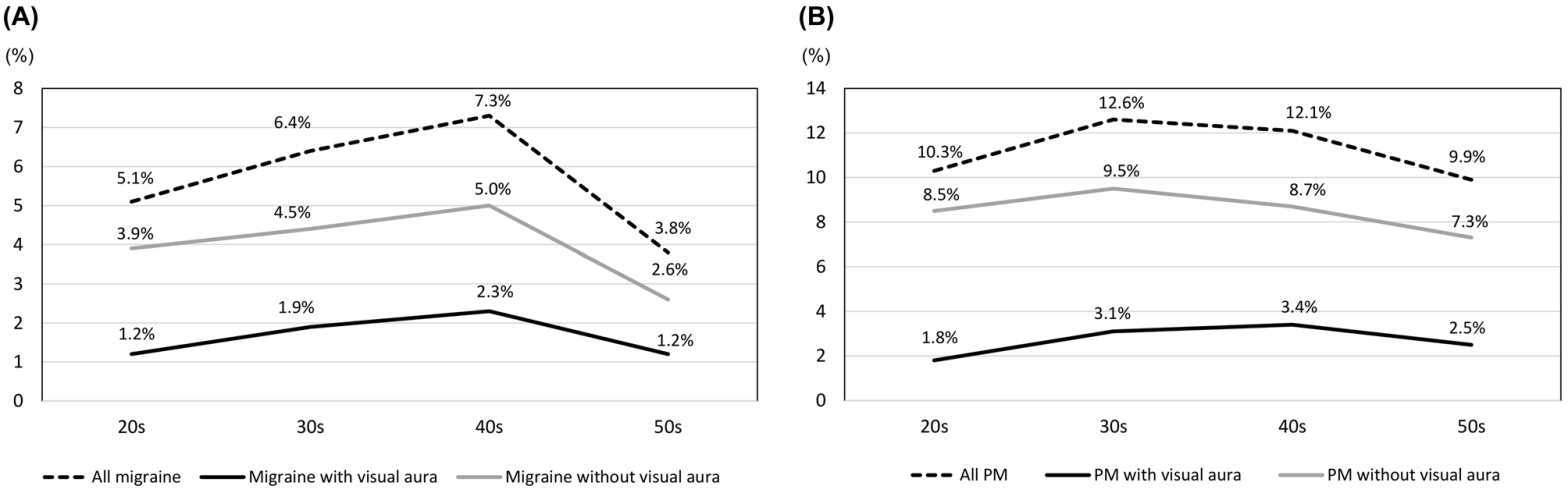
Age-specific prevalence of visual aura in migraine (**A**) and probable migraine (**B**). PM: probable migraine.

The age-specific prevalence of migraine with VA, migraine without VA, and overall migraine showed similar trends. The highest prevalence of all three was seen in participants in their 40s, followed by in participants in their 30 s; the lowest prevalence was noted in participants in their 50s (Fig. 3A). The prevalence of PM with VA was also the highest in those in their 40s, followed by in those in their 30s (Fig. 3B). However, the prevalence of PM with VA (*p* = 0.232) and of PM without VA (*p* = 0.466) did not differ statistically significantly with the age groups.

### Clinical characteristics of migraine and PM according to the presence of VA

The number of headache days per month (*p* = 0.044), HIT-6 scores (*p* = 0.046), ASC-12 scores (CA; *p* = 0.043), BEPSI-K scores (stress; *p* = 0.044), and rate photophobia (*p* = 0.038) were statistically significantly higher in participants with migraine with VA than in participants with migraine without VA (Table 3).

**Table 3:** Demographic and clinical characteristics according to the presence or absence of visual aura in migraine and probable migraine.

	Migraine with visual aura, N = 50	Migraine without visual aura, N = 120	*P* value	PM with visual aura, N = 82	PM without visual aura, N = 255	*P* value
Age (years)	40.5 (33.8–47.5)	40.0 (31.3–45.8)	0.487	43.5 (33.0–50.0)	40.0 (30.0–49.0)	0.113
Women	32 (64.0)	97 (80.8)	0.019	46 (56.1)	161 (63.1)	0.255
HA duration in hours	12.0 (6.4–40.0)	20.0 (6.0–48.0)	0.456	0.1 (0.1–0.2)	0.1 (0.1–0.2)	0.092
Frequency	4.0 (2.0–10.0)	2.0 (1.0–4.8)	0.320	2.0 (2.0–8.0)	2.0 (0.6–4.0)	0.006
HA days per month	4.0 (2.0–10.0)	3.0 (2.0–5.0)	0.044	3.0 (2.0– 5.0)	2.0 (1.0–4.0)	0.007
Severe HA days per month	2.50 (1.0–5.0)	2.00 (1.0–3.0)	0.060	2.0 (1.0–3.0)	1.0 (1.0–2.0)	0.004
HA days with medication	3.00 (1.0–5.3)	2.00 (1.0–5.0)	0.051	2.0 (1.0–5.0)	1.0 (0.0–2.0)	< 0.001
Severe HA intensity	21 (42.0)	58 (48.3)	0.622	0 (0.0)	11 (4.3)	0.997
Moderate-to-severe intensity	50 (100.0)	120 (100.0)	1.000	55 (67.1)	172 (67.4)	0.949
VAS score	7.0 (7.0–8.0)	7.00 (7.0–8.0)	0.327	6.0 (4.0–7.0)	6.0 (4.0–7.0)	0.604
HIT-6 score	53.0 (46.0–60.3)	50.0 (45.0–56.8)	0.046	49.5 (44.0–56.0)	50.0 (44.0–58.0)	0.509
MIDAS score	14.5 (8.8–32.3)	12.5 (5.0–27.8)	0.163	10.0 (5.0–26.3)	5.0 (2.0–12.0)	< 0.001
Unilateral pain	22 (44.0)	69 (57.5)	0.130	60 (72.3)	181 (70.7)	0.937
Pulsating pain	34 (68.0)	73 (60.8)	0.529	53 (64.6)	196 (76.9)	0.018
Aggravation by movement	40 (80.0)	98 (81.7)	0.971	56 (68.3)	128 (50.2)	0.082
Nausea	31 (62.0)	89 (74.2)	0.520	69 (84.1)	213 (83.5)	0.967
Vomiting	24 (48.0)	58 (48.3)	0.577	43 (52.4)	89 (34.9)	0.011
Photophobia	42 (84.0)	83 (69.2)	0.038	48 (58.5)	98 (38.4)	0.001
Phonophobia	43 (86.0)	94 (78.3)	0.087	53 (64.6)	113 (44.3)	< 0.001
WPI	5.0 (3.0–7.0)	4.0 (2.0–6.0)	0.195	5.0 (4.0–7.0)	3.0 (2.0–6.0)	0.001
FS	12.0 (10.0–15.0)	12.0 (8.0–14.0)	0.032	12.0 (9.8–13.3)	9.0 (6.0–12.0)	< 0.001
PHQ-9 score	8.0 (7.0–9.0)	8.0 (5.0–9.0)	0.755	9.0 (6.0–10.3)	7.0 (4.0–9.0)	< 0.001
GAD-7 score	8.0 (3.8–11.5)	5.0 (3.0–9.0)	0.085	7.0 (4.0–11.0)	4.0 (1.0–7.0)	< 0.001
ASC-12 score	3.0 (1.0–8.0)	2.0 (0.0–4.7)	0.043	2.5 (0.0–6.0)	0.0 (0.0–3.0)	< 0.001
BEPSI-K score	2.6 (2.0–3.2)	2.2 (1.8–3.0)	0.044	2.3 (2.0–3.0)	2.0 (1.0–2.8)	0.001
M-TOQ-6 score	20.5 (16.3–24.0)	22.0 (18.0–26.0)	0.051	21.0 (17.3–25.0)	23.0 (21.0–26.5)	0.003

PM: probable migraine, HA: headache, VAS: visual analogue scale, HIT-6: Headache Impact Test-6, MIDAS: Migraine Disability Assessment, WPI: Widespread Pain Index, FS: Fibromyalgia symptom severity score, PHQ-9: Patient Health Questionnaire-9, GAD-7: Generalized Anxiety Disorder-7, ASC-12: 12-item Allodynia symptom checklist, BEPSI-K: Korean version of the Brief Encounter Psychosocial Instrument, M-TOQ-6: 6-item version of the Migraine-Treatment Optimization Questionnaire.

For PM, the headache frequency per month in participants with VA was statistically significantly higher than in participants without VA (*p* = 0.004). Furthermore, CA (*p* < 0.001), stress (*p* = 0.001), anxiety (GAD-7; *p* < 0.001), depression (PHQ-9; *p* < 0.001), and widespread pain (WPI; *p* = 0.001) were more severe in participants having PM with VA than in those having PM without VA. The response to acute medications (M-TOQ-6) was less in participants having PM with VA than in those having PM without VA (*p* = 0.003). Vomiting (*p* = 0.011), photophobia (*p* = 0.001), and phonophobia (*p* < 0.001) were more prevalent in participants having PM with VA than in those having PM without VA (Table 3).

### Clinical characteristics of migraine and PM according to the frequency of VA in participants with migraine and PM

We dichotomized the participants with migraine with VA and those with PM with VA according to the occurrence of VA in 51% of the headache attacks (set as the cut-off). Accordingly, 13 (26%) and 37 (74.0%) participants with migraine experienced VA in ≥ 51% and < 51% of all headache attacks, respectively. Among the participants with PM, 15 (18.3%) and 67 (81.7%) experienced VA in ≥ 51% and < 51% of all headache attacks, respectively.

Participants with migraine who experienced VA in ≥ 51% of all headache attacks experienced a higher stress than those who experienced VA in < 51% of all headache attacks (*p* = 0.045). Furthermore, compared to participants with migraine with VA in < 51% of all headache attacks, those with migraine with VA in ≥ 51% of all headache attacks comprised more men (*p* = 0.004). Other clinical characteristics did not differ statistically significantly between the two (Table 4).

**Table 4:** Comparison of the demographic and clinical characteristics of the participants (with migraine and probable migraine) presenting with visual aura in < 51% and > 51% of all headache attacks.

	Migraine with visual aura in ≥ 51% of all headache attacks, N = 13	Migraine with visual aura in < 51% of all headache attacks, N = 37	*P* value	PM with visual aura in ≥ 51% of all headache attacks, N = 15	PM with visual aura in < 51% of all headache attacks, N = 67	*P* value
Age (years)	38.0 (34.0–45.0)	41.0 (33.0–49.0)	0.595	37.0 (30.0–46.0)	45.0 (33.0–50.0)	0.117
Women	4 (30.8)	28 (75.7)	0.004	10 (66.7)	36 (53.7)	0.404
Frequency	2.0 (0.75–8.0)	4.0 (2.0–10.0)	0.719	4.0 (1.0–8.0)	2.0 (2.0–8.0)	0.555
HA days per month	2.0 (1.0–10.0)	5.0 (2.0–10.0)	0.282	3.0 (1.0–5.0)	2.0 (2.0–5.0)	0.336
Severe HA days per month	2.0 (1.0–5.0)	3.0 (1.0–5.5)	0.381	3.0 (1.0–5.0)	2.0 (1.0–3.0)	0.876
HA days with medication	1.0 (0.0–15.0)	3.0 (1.0–5.0)	0.518	2.0 (0.0–5.0)	2.0 (1.0–5.0)	0.615
Severe HA intensity	5 (38.5)	16 (43.2)	0.847	0 (0.0–0.0)	0 (0.0–0.0)	1.000
VAS score	7.0 (7.0–8.0)	7.0 (7.0–8.0)	0.655	7.0 (4.0–8.0)	6.0 (4.0–7.0)	0.455
HIT-6 score	50.0 (44.0–53.0)	54.0 (46.5–61.0)	0.545	46.0 (41.0–55.0)	50.0 (44.0–56.0)	0.328
MIDAS score	11.0 (6.0–41.5)	15.0 (9.0–15.0)	0.417	11.0 (5.0–47.0)	10.0 (5.0–24.0)	0.200
Unilateral pain	6 (46.2)	16 (43.2)	0.884	9 (60.0)	51 (76.1)	0.303
Pulsating pain	8 (61.5)	26 (70.3)	0.275	9 (60.0)	44 (65.7)	0.752
Aggravation by movement	11 (84.6)	29 (78.4)	0.215	12 (80.4)	44 (65.7)	0.268
Nausea	9 (69.2)	22 (59.5)	0.574	12 (80.0)	57 (85.1)	0.702
Vomiting	9 (69.2)	15 (40.4)	0.401	6 (40.0)	37 (55.2)	0.481
Photophobia	13 (100.0)	29 (78.4)	0.998	12 (80.0)	36 (53.7)	0.071
Phonophobia	9 (69.2)	22 (59.5)	0.121	11 (73.3)	42 (62.7)	0.567
WPI	3.0 (1.0–6.0)	6.0 (4.0–7.5)	0.559	7.0 (4.0–9.0)	4.0 (3.0–7.0)	0.028
PHQ-9 score	9.0 (7.5–10.5)	8.0 (6.0–9.0)	0.400	10.0 (8.0–18.0)	8.0 (6.0–9.0)	0.016
GAD-7 score	9.0 (4.5–10.5)	8.0 (3.0–13.0)	0.754	12.0 (5.0–15.0)	6.0 (4.0–9.0)	0.013
ASC-12 score	4.0 (2.5–8.0)	3.0 (0.5–8.0)	0.675	4.0 (2.0–8.0)	2.0 (0.0–6.0)	0.572
BEPSI-K score	3.0 (2.7–3.8)	2.6 (2.0–3.0)	0.045	3.2 (2.2–4.0)	2.2 (2.0–2.8)	0.006
M-TOQ-6 score	20.5 (15.3–22.8)	20.0 (16.5–24.3)	0.555	21.0 (18.0–26.0)	21.0 (17.0–25.0)	0.827
PSQI	9.0 (7.5–10.0)	7.0 (5.5–9.0)	0.130	10.0 (7.0–10.0)	7.0 (5.0–9.0)	0.032

PM: probable migraine, HA: headache, VAS: visual analogue scale, HIT-6: Headache Impact Test-6, MIDAS: Migraine Disability Assessment, WPI: Widespread Pain Index, PHQ-9: Patient Health Questionnaire-9, GAD-7: Generalized Anxiety Disorder-7, ASC-12: 12-item Allodynia symptom checklist, BEPSI-K: Korean version Brief Encounter Psychosocial Instrument, M-TOQ-6: 6-item version of the Migraine-Treatment Optimization Questionnaire, PSQI: Pittsburgh Sleep Quality Index.

Among the participants with PM with VA, those with VA in ≥ 51% of all headache attacks had more widespread pain (*p* = 0.028), depression (*p* = 0.016), anxiety (*p* = 0.013), stress (*p* = 0.006), and poorer sleep quality (*p* = 0.032) as compared with those with VA in < 51% of all headache attacks (Table 4).

### Clinical characteristics of migraine with VA and PM with VA

We further compared the demographic and clinical characteristics between those with migraine with VA and those with PM with VA. Participants with migraine with VA had a more severe headache intensity (VAS score; *p* < 0.001), lesser unilateral pain (*p* < 0.001), lesser nausea (*p* = 0.004), and greater photophobia (*p* = 0.002) and phonophobia (*p* = 0.008) than participants with PM with VA (Supplementary Table 1).

## Discussion

The main findings of the present study are as follows: (1) in a general population-based sample, 29.4% of the participants with migraine and 24.3% of the participants with PM experienced VA during the previous year; (2) the female-to-male ratio for the prevalence of migraine with VA was significantly lower than that for the prevalence of migraine without VA and the female-to-male ratios for the prevalence of PM with VA and PM without VA did not differ significantly; and (3) some symptoms of migraine and PM were more severe when accompanied with VA.

Traditionally, four types of migraine auras have been reported: VA, sensory aura, language aura, and motor aura^33^. These auras may appear alone or in combination with other auras. VA is the most common type (98% of the cases with MA), followed by sensory aura. Language and motor auras are uncommon^3,4^. Brainstem and retinal auras, which are rare, have also been included in ICHD-1^18^. Given that VA presents in most individuals with MA and that the self-administered VARS has a high sensitivity for detecting VA, we assumed that most participants with MA were identified as having migraine with VA in the present study^12^.

Several studies have reported the prevalence of MA in migraine populations. The American Migraine Survey II, a nation-wide population study in the United States, reported that MA presented in 31% of the migraine population^34^. An epidemiological study in Copenhagen, Denmark, demonstrated that 33.7% of the individuals with migraine were classified as having an aura^6^. In a German nation-wide study, the prevalence of MA among individuals with migraine was 34.0%^35^. However, studies in Asian countries revealed a somewhat lower proportion of MA in the migraine population. A population-based survey in Taipei, Taiwan, reported a prevalence of 12.5%, while a Malaysian community study reported a prevalence of 10.6%^9,36^.

Previous studies on the prevalence of MA used questionnaires that were not specially validated for aura detection. To the best of our knowledge, the present study is the first to use a validated instrument for VA. The prevalence of MA in the present study was 30.3%, which is similar to that reported in previous studies in the United States and European countries. Possible causes of the lower proportion of MA among migraine populations in previous Asian studies, as compared to in American and European studies, include differences in the ethnicity, lifestyle, and assessment methodology.

Although female predominance was found both in MA and MO, the female-to-male ratio for the prevalence of MA was significantly lower than that for the prevalence of MO in previous studies. The female-to-male ratio for the lifetime prevalence of MA was 1.5, whereas the female-to-male ratio for the prevalence of MO was 2.2 in a Danish population-based study^6^. A Taiwanese study reported female-to-male ratios of 2.6 and 4.2 for the prevalence of MA and MO, respectively^9^. The present study found similar results: female predominance was noted in migraine with VA and in migraine without VA. However, the female-to-male ratio for the prevalence of migraine with VA (1.86) was significantly lower than that for the prevalence of migraine without VA (4.42). Lower female predominance in MA than in MO indicates differences in the roles of sex hormones between the pathogeneses of MA and MO^37^. A difference in the occurrence of attacks between MA and MO according to the female hormonal cycle was also reported; the study suggested that estrogen withdrawal was associated with attacks of MO and high plasma estrogen was associated with the occurrence of MA^37^.

The female-to-male ratio for the prevalence of PM, according to the presence of aura, has rarely been reported. This study found that female predominance in the prevalence of PM was less prominent as compared to that in the prevalence of migraine, and the female-to-male ratios did not differ significantly with the presence or absence of VA. Therefore, findings regarding the female-to-male ratios for the prevalence of PM propose different roles of sex hormones in the pathogenesis of PM^11,38^.

The present study found a higher frequency of headache and more severe CA in participants with migraine with VA than in those with migraine without VA. One possible explanation for the differences in the clinical presentation of migraine with respect to the presence or absence of VA is the difference in the mechanism of migraine between the two. Cortical spreading depression (CSD) has been considered a key mechanism of MA^40,41^. Although there are some controversies^42^, the role of CSD in MO is not well understood^43,44^. Our study findings suggest that CSD might be involved in some migraine attacks in individuals with migraine with VA; this may be responsible for the differences in some clinical characteristics between participants with migraine with VA and those with migraine without VA.

The MIDAS scores of participants with PM with VA were higher than those of participants with PM without VA. Conversely, the MIDAS scores of participants with migraine with VA did not differ significantly from those of participants with migraine without VA. This discrepancy may be due to differences in the severity of the symptoms and disability between migraine and PM. The symptoms and disability of participants with PM were milder than those of participants with migraine^14,45^. Because the symptoms of migraine were severe enough, an exacerbation of some symptoms with the accompaniment of VA did not affect disability. In PM, which was associated with milder disability, the worsening of symptoms due to VA accompaniment may have exacerbated disability significantly.

The present study has several limitations. First, the cooperation rate of the present study was not high at 28.3%. Such a low cooperation rate in a web-based survey has already been reported. The Chronic Migraine Epidemiology and Outcomes study was a nation-wide web-based survey for migraine in the USA, and its cooperation rate (16.5%) was lower than that of the American Migraine Prevalence and Prevention survey (65.8%), a traditional mailed survey^46^. Nevertheless, the distributions of age, sex, size of residential area, and educational level in our sample were not significantly different from those of the total population of Korea. In addition, the prevalence of migraine (5.6%) and PM (11.3%) in our study were similar to those reported in previous studies in Korea (4.7%–9.1%) and other Asian countries (6.1%–12.8%)^9,10,36,38^. Second, although we used data from a nation-wide population-based study with a large sample size, its external validity is needed for the generalizability of our findings. Common issues that limit external validity include selection biases, aptitude, treatment interactions, and so on^47^. Therefore, the findings of the present study should be verified in various populations.

The strengths of the present study are as follows. First, we used a validated instrument to assess the presence of VA. Previous epidemiological studies evaluated aura symptoms using questionnaires that were not validated for the detection of aura. The present study used the self-administered VARS questionnaire, which has been validated by comparing its results to a doctor’s diagnosis of VA^12^. Second, we evaluated the prevalence and impact of VA in PM in addition to that in migraine. PM is a prevalent headache disorder that fulfils all but one criterion of migraine. Yet, the prevalence and impact of VA have rarely been documented.

In conclusion, the present study evaluated the prevalence and impact of VA in migraine and PM in a general population-based sample using a validated instrument. The prevalence of VA was not significantly different between migraine and PM. Some symptoms and comorbidities of migraine and PM with VA were more severe as compared with those of migraine and PM without VA.

## Supplementary Information

Supplementary Information.

## Data Availability

Anonymized data relevant to this study will be shared upon request with a qualified investigator after appropriate institutional review board approvals.
