# Supplementary material for: Prevalence and impact of visual aura in migraine and probable migraine: a population study
Source: Sci Rep. 2022 Jan 10;12:426. doi: 10.1038/s41598-021-04250-3 (PMC8748892; doi:10.1038/s41598-021-04250-3)
Supplement: Supplementary file 1 — Supplementary Information. [file 41598_2021_4250_MOESM1_ESM.docx]

**Prevalence and impact of visual aura in migraine and probable migraine: A population study**

Kyung Min Kim^1*^, Byung-Kun Kim^2*^, Wonwoo Lee^1^, Heewon Hwang^1^, Kyoung Heo^1^, Min Kyung Chu^1^

**Supplementary Table 1.** Migraine with visual aura vs. probable migraine with visual aura

|  | Migraine with visual aura, N=50 | Probable migraine with visual aura, N=82 | P-value |
| --- | --- | --- | --- |
| Age | 40.00 (33.75-47.50) | 43.50 (33.00-50.00) | 0.465 |
| Women | 32 (64.0) | 46 (56.1) | 0.370 |
| Frequency | 4.0 (2.0-10.0) | 2.0 (2.0-8.0) | 0.157 |
| HA duration (hr) | 12.00 (6.40-40.04) | 0.08 (0.08-0.16) | <0.001 |
| HA days per month | 4.0 (2.0-10.0) | 3.0 (2.0-5.0) | 0.060 |
| Severe HA days per month | 2.50 (1.00-5.00) | 2.0 (1.0-3.0) | 0.259 |
| HA days with medication | 3.00 (1.00-5.25) | 2.0 (1.0-5.0) | 0.639 |
| Severe intensity | 21 (42.0) | 0 (0.0) | <0.001 |
| Moderate-to-severe intensity | 50 (100.0) | 54 (65.9) | <0.001 |
| VAS | 7.00 (7.00-8.00) | 6.0 (4.0-7.0) | <0.001 |
| MIDAS | 14.50 (8.75-32.25) | 10.00 (5.00-26.25) | 0.061 |
| HIT-6 | 53.00 (46.00-60.25) | 49.50 (44.00-56.00) | 0.053 |
| ASC-12 | 3.00 (1.00-8.00) | 2.50 (0.00-6.00) | 0.274 |
| WPI | 5.00 (3.00-7.00) | 5.00 (3.75-7.00) | 0.697 |
| Unilateral | 22 (44.0) | 60 (73.2) | 0.001 |
| Pulsating | 34 (68.0) | 53 (64.6) | 0.692 |
| Aggravation by movement | 40 (90.0) | 56 (68.3) | 0.143 |
| Nausea | 31 (62.0) | 69 (84.1) | 0.004 |
| Vomiting | 24 (48.0) | 43 (52.4) | 0.621 |
| Photophobia | 42 (84.0) | 48 (58.5) | 0.002 |
| Phonophobia | 43 (86.0) | 53 (64.6) | 0.008 |
| GAD-7 | 8.00 (3.75-11.50) | 7.00 (4.00-11.000) | 0.771 |
| PHQ-9 | 8.00 (7.00-9.00) | 9.00 (6.00-10.25) | 0.478 |
| BEPSI-K | 2.60 (2.00-3.20) | 2.30 (2.00-3.00) | 0.118 |
| PSQI | 8.00 (6.00-9.25) | 7.00 (5.00-10.00) | 0.593 |

HA: headache, VAS: visual analogue scale, MIDAS: Migraine Disability Assessment, HIT-6: Headache Impact Test-6, ASC-12: 12-item Allodynia symptom checklist, WPI: Widespread Pain Index, GAD-7: Generalized Anxiety Disorder-7, PHQ-9: Patient Health Questionnaire-9, BEPSI-K: Korean version Brief Encounter Psychosocial Instrument, PSQI: Pittsburgh Sleep Quality Index
